# DraGnET: Software for storing, managing and analyzing annotated draft genome sequence data

**DOI:** 10.1186/1471-2105-11-100

**Published:** 2010-02-22

**Authors:** Stacy Duncan, Ruchita Sirkanungo, Leslie Miller, Gregory J Phillips

**Affiliations:** 1 Department of Veterinary Microbiology and Preventive Medicine, Iowa State University, Ames, Iowa, USA; 2 Department of Computer Science, Iowa State University, Ames, Iowa, USA; 3 Interdepartmental Bioinformatics and Computational Biology, Iowa State University, Ames, Iowa, USA

## Abstract

**Background:**

New "next generation" DNA sequencing technologies offer individual researchers the ability to rapidly generate large amounts of genome sequence data at dramatically reduced costs. As a result, a need has arisen for new software tools for storage, management and analysis of genome sequence data. Although bioinformatic tools are available for the analysis and management of genome sequences, limitations still remain. For example, restrictions on the submission of data and use of these tools may be imposed, thereby making them unsuitable for sequencing projects that need to remain in-house or proprietary during their initial stages. Furthermore, the availability and use of next generation sequencing in industrial, governmental and academic environments requires biologist to have access to computational support for the curation and analysis of the data generated; however, this type of support is not always immediately available.

**Results:**

To address these limitations, we have developed DraGnET (Draft Genome Evaluation Tool). DraGnET is an open source web application which allows researchers, with no experience in programming and database management, to setup their own in-house projects for storing, retrieving, organizing and managing annotated draft and complete genome sequence data. The software provides a web interface for the use of BLAST, allowing users to perform preliminary comparative analysis among multiple genomes. We demonstrate the utility of DraGnET for performing comparative genomics on closely related bacterial strains. Furthermore, DraGnET can be further developed to incorporate additional tools for more sophisticated analyses.

**Conclusions:**

DraGnET is designed for use either by individual researchers or as a collaborative tool available through Internet (or Intranet) deployment. For genome projects that require genome sequencing data to initially remain proprietary, DraGnET provides the means for researchers to keep their data in-house for analysis using local programs or until it is made publicly available, at which point it may be uploaded to additional analysis software applications. The DraGnET home page is available at http://www.dragnet.cvm.iastate.edu and includes example files for examining the functionalities, a link for downloading the DraGnET setup package and a link to the DraGnET source code hosted with full documentation on SourceForge.

## Background

DNA sequencing technology using chain-terminating dideoxy nucleoside triphosphates, first developed by Frederick Sanger [1,2], has remained the mainstay of genome sequencing efforts for more than thirty years. However, recently developed, new massively parallel DNA sequencing platforms are now extensively used to generate sequence data at a fraction of the cost and labor required by Sanger technology. Three "next generation" sequencing systems that are currently commercially available include the Roche/454 Genome Sequencer [3], Illumina/Solexa Genome Analyzer II [4,5] and Applied Biosystems SOLiD System [6]. In addition, commercial release of two additional platforms, including the Helicos Heliscope and the Pacific Biosmart SMRT, are planned for 2010 [7].

Collectively, these systems, with their high depth of coverage and relatively low costs, have allowed individual researchers to initiate genome sequencing projects that were previously available to only large genome centers [8-10]. The enhanced sequencing capability afforded by next-generation sequencing has had an especially significant impact on bacterial genomics. By facilitating genome sequencing of multiple isolates of the same bacterial species, several examples of extensive intraspecies genotypic heterogeneity have been revealed, leading to a revision of many long-standing views of microbial speciation [11-14]. One of the first such studies revealed significant genetic variability among eight different strains of *Streptococcus agalactiae*, group B *Streptococcus *(GBS) [14]. After performing cross strain comparisons Tettelin *et al*. found a considerable number of genes not shared among the strains. Their discovery led to the proposal of the bacterial "pan-genome", defined as the global gene repertoire of a bacterial species comprised of the core genome (the set of genes shared by all the strains of the same bacterial species), the dispensable genome (the set of genes present in some but not all of the strains) and the strain specific genes (the set of genes found only in a single strain) [14]. Genome heterogeneity has also been noted for species of *Helicobacter pylori*, *Staphylococcus aureus*, and *Escherichia coli *[13,15,16]. As noted by Muzzi *et al*., comparative genomics of bacterial species has important implications for vaccine development and discovery of novel antimicrobials [17]. Other novel applications for next generation sequencing technologies have also been developed, including bacterial metagenomics [18-20], and transcriptome mapping [21-24].

Despite the potential for new insights into bacterial diversity and function, important challenges continue to include the organization, management and analysis of genome sequencing data. To address the need for tools for querying, analyzing and comparing multiple genomes of related species, several databases and software tools have been developed [25], including the Integrated Microbial Genomes (IMG) system [26,27], Integrated Microbial Genomes-Expert Review (IMG ER) system [28], GenColors [29,30], the Microbial Genome Database (MBGD) [31,32], the Comprehensive Microbial Resource (CMR) [33] and the EDGAR software [34].

The IMG system contains complete and draft microbial genome sequence data generated by the Joint Genomes Institute (JGI) as well as other publicly available genome data not limited to microorganisms. Tools provided through IMG allow users to query, view and perform comparative analysis of genomes, genes and functions. Recently, a new version of IMG called IMG ER has been added to the IMG system. Tools available through IMG ER allow users to analyze and curate annotated microbial genome data whether it is unpublished or published. Although IMG ER allows users to upload their genome sequencing data for curation and analysis, it is not available for download and in-house use. The GenColors software allows users to browse, analyze and compare genome information from complete and ongoing genome projects related to prokaryotic or eukaryotic genomes. Additionally, GenColors may be used for the purpose of annotation in the case of incomplete projects. The CMR software contains sequence and annotation data for all of the current publicly available completed microbial genomes and provides a variety of comparison tools for the analysis of the multiple genomes including cross-genome analysis capabilities. Currently, however, there is no functionality that allows users to submit genome data for use with CMR. Similar to CMR, MBGD provides users with several tools for the comparison and analysis of complete bacterial genomes. Unlike CMR, MBGD contains a newly added feature called MyMBGD that allows users to add their own genome data to MBGD. The EDGAR software has recently been released and includes comparative analysis tools for the comparison of multiple strains of a given species. EDGAR offers similar capabilities to those found in CMR and MBGD, in addition to features such as phylogenetic analyses and visualization capabilities including Venn diagrams and synteny plots.

While the aforementioned systems include data management and analysis functionalities there are limitations. For example, genome projects that include proprietary data may be restricted in the submission of the data to third party software. Many of the current data management software tools are not available for download and in-house use, a requirement when access to next generation sequencing instruments can outstrip the availability of experienced bioinformaticians to assist with data management and analysis.

In addition to the already mentioned software applications, there are other tools that are designed for genome annotation or re-annotation of unpublished or published genomes [25,35,36]. Several of these tools provide data curation capabilities for the purpose of correcting annotation errors and improving annotated data but are restricted to use with the annotated data generated through specified software packages. Additionally, as with many software applications, they require the researcher to develop a working knowledge of the analysis capabilities of the software as well as provide "expert" curation of the data. With the increased use of next-generation sequencing in academic, industrial and government settings, however, biologists do not always have immediate access to computational support needed to easily manage the data and to initiate comparative analysis.

To overcome some of these limitations, DraGnET was developed specifically to provide biologists with their own web based tool that is both convenient and easy to use. DraGnET allows researchers to independently store, retrieve and curate their own data generated from any annotation engine and to perform genome comparisons during the beginning phase of a sequencing project. Additionally, publicly available genome data can be stored for the purpose of comparing draft genome data with reference genomes. DraGnET includes provisions for data access, searching, and modification as well as access to basic local alignment search tool (BLAST) functionalities [37] for amino acid sequence similarity searches and cross strain comparisons. As a consequence, DraGnET allows investigators to immediately begin testing of biologically relevant hypotheses without having to devote time to learning sophisticated analysis programs or to depend on computational support from designated personnel. Additionally, the DraGnET source code has been made available, allowing researchers to further customize and develop the software to meet the needs of specific sequencing projects.

To demonstrate the utility of DraGnET, we have successfully established a DraGnET project, deployed for Internet access, and performed preliminary cross strain comparisons to identify potential vaccine targets against the animal pathogen *Haemophilus parasuis*. Microbial genome sequencing has proven to be a powerful approach to identify new, protective vaccines via *reverse vaccinology*, i.e., discovery of vaccine targets by scanning sequence data for potential surface-exposed antigens [38]. Moreover, broadly protective antigens may be identified by comparison of genomes from multiple strains of a single species [17,39,40]. Reverse vaccinology has led to the development of new vaccines for several human and animal pathogens where previously vaccines were not available [41-44]. DraGnET enables facile preliminary comparisons of multiple draft or complete genome sequences of any number of organisms, including identification of protein encoding genes shared by multiple strains, making DraGnET a useful bioinformatic tool.

## Implementation

**Figure 1:**
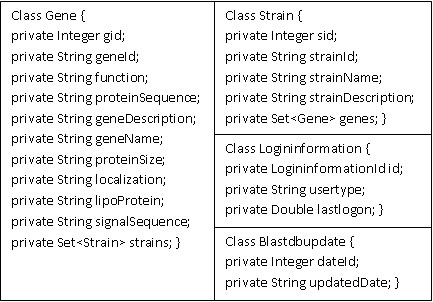
**Java classes**. Four Java classes are used to define the Gene, Strain, Logininformation and Blastdbupdate objects. The Gene class defines variables for the following annotated gene information: gene identification (gid and geneId), gene function (function), the protein sequence (proteinSequence), a description of the gene (geneDescription), the name of the gene (geneName), the size of the protein (proteinSize), the subcellular localization (localization), if the protein is predicted to be a lipoprotein (lipoprotein), if the protein is predicted to have a signal sequence (signalSequence) and the set of strains that contain the genes (Set<Strain> strains). The Strain class defines variables for strain information such as a strain identifier (sid and strainId), the strain name (strainName), a description of the strain (strainDescription), and the set of genes contained in the strain (Set<Gene> genes). The Logininformation class defines variables for the user login identifier (LogininformationId id), the usertype and the time the user logged in (lastlogon). The Blastdbupdate class defines variables for the date the last update was made to the data (dateId and updatedDate).

**Figure 2:**
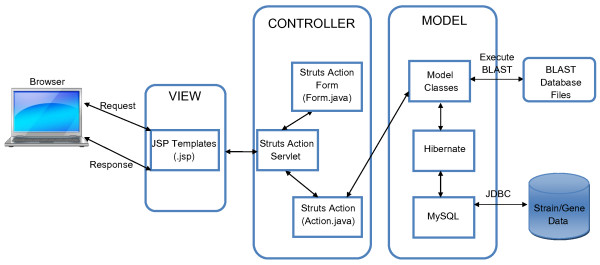
**DraGnET software architecture**. The DraGnET web application uses Struts to implement the Model-View-Controller (MVC) architecture. The view represents the presentation of the application and is implemented through Java Server Pages (JSP). The Controller is responsible for intercepting and translating user input into actions to be performed by the Model. The Controller receives the request from the browser, invokes a business operation and coordinates the view to be returned to the browser. The Struts Action servlet populates information from the JSP to the appropriate Struts Action Form then throws control to the Struts Action. The Struts Action gets data from the appropriate Struts Action Form and sends the information to the Model where certain actions like retrievals and updates will be performed. The Model is where communication with the database takes place through Hibernate. Hibernate is used to map Model Classes (Java objects) to tables in the database. Model Classes are also used to execute BLAST functionalities provided through the application's web interface. The Model represents enterprise data and the business rules that govern access to and updates of this data.

The DraGnET web application was developed in Java [see Additional file 1]. DraGnET provides user interfaces for storing information related to strains and their associated annotated gene set in a database. Gene and strain information are stored as objects defined by two Java classes, Gene and Strain (Figure 1). The Gene class stores nine gene attributes most of which can be obtained from gene annotation data. The choice of gene attributes was based upon gene information available in public sequence databases such as GenBank and includes additional attributes relevant for vaccine target identification. The Strain class contains information such as the strain name and description. Two additional Java classes, Logininformation class and the Blastdbupdate class are used to define objects related to administrator/curator user information and the date of the last modification made to the data, respectively (Figure 1). Hibernate (version 3.1 core and advanced libraries) is used to map the Java objects, representing the Gene, Strain, Logininformation and Blastdbupdate classes, to relational tables in a MySQL (version 5.0) database. By using Hibernate in the software architecture, DraGnET works with an object database supported by Hibernate. The servlet engine used to support the web interface is Apache-Tomcat version 6. The web application uses Struts (version 1.2) to implement the Model-View-Controller (MVC) architecture. The MVC architecture provides a way to separate the web interface (view) from the business logic (model) making it easier to implement and modify either component independent of the other. The web interface (view) is implemented through Java Server Pages (JSP). BLAST functionalities are provided by stand-alone executable BLAST software connected to the business logic and web pages are provided for users to interact with BLAST. The BLAST program is configured to run the blastp (protein blast) algorithm and applies the blastall program available from NCBI [45]. The general layout for the architecture of the DraGnET software is provided in Figure 2. The web application was built using MyEclipse version 6.0 and has been successfully tested on Microsoft Windows 2003 and Windows XP operating systems.

### DraGnET project setup

**Figure 3:**
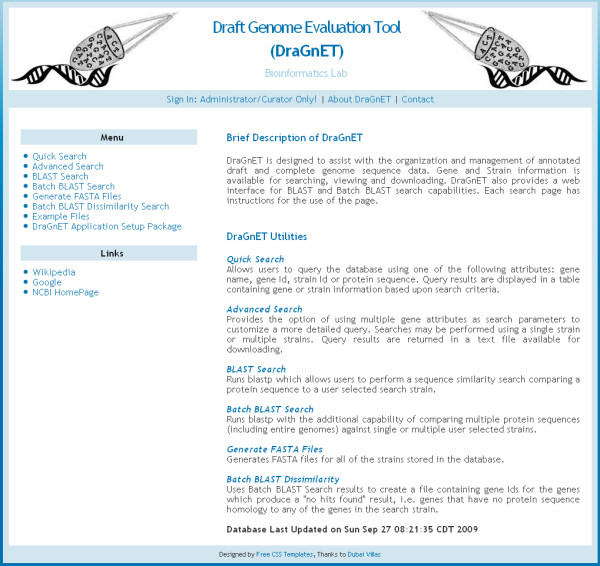
**Web interface- DraGnET Home Page**. Listed on the DraGnET home page are links for downloading the DraGnET setup package ("DraGnET Application Setup Package"), testing search and BLAST capabilities ("Example Files"), generating FASTA formatted files ("Generate FASTA files") and all "Search" functionalities.

A DraGnET project can be installed on a personal computer or it can be setup for Internet (or Intranet) deployment making it a tool that is available for collaborative projects. The initial setup of a DraGnET project requires installation of Java (version 1.6), MySQL (version 5.0) including the MySQL 5.0 GUI Tools, Apache Tomcat 6, and Blast 2.2.18. Executable files for installing all of the aforementioned software are provided in a comprehensive setup package provided through the "DraGnET Application Setup Package" link located on the application's home page (Figure 3). After installing the required software packages the database structure used by Hibernate to map the Java objects to relational tables in the MySQL database is automatically generated by the MySQL 5.0 GUI Tool and a file included in the setup package [see Additional file 2]. This automated process alleviates the requirement of the user to have the knowledge necessary for setting up the database schema used to store the genome data. After the DraGnET project is set up and genome sequence data has been uploaded into the database, local BLAST databases for each genome need to be formatted for use with the BLAST functionalities provided with the application. Information on all of these steps, as well as additional usage information, is available in the DraGnET_setup.doc provided in the setup package.

## Results

DraGnET is an open source web application designed to provide researchers with a tool for storing their own unpublished annotated draft and complete genome data from multiple strains of a species in a database; allowing gene and strain information to be available for retrieving, searching, modifying and downloading. The application also provides a web interface for the use of BLAST, allowing for protein sequence similarity searches and cross strain comparisons of strains stored in the database. In addition, DraGnET provides a link for the automatic generation of FASTA files for each genome stored in the database. The files are available for download and can be used with other software and tools for further analysis. The details of the functionalities of DraGnET are provided in the following section.

### Data Management

DraGnET is set up to allow any user to search, view and compare genome sequence data stored in the database; however, only curators may insert and modify the data by signing in to the application. This was designed to prevent inconsistencies in the data and to protect the application when it is being accessed by multiple users from different locations.

#### Data insertion

**Figure 4:**
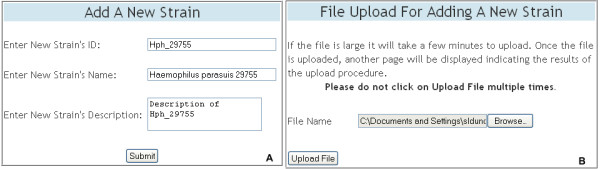
**Adding a new strain**. The data entry tables displayed on the web pages for inserting a new strain. In the first table (A) the curator enters the strain Id, strain name and strain description of the new strain. In the second table (B) the curator is directed to upload a file containing gene information for genes contained in the strain. The strain and gene information is then stored in the database.

Two web pages are provided for the insertion of strain and gene information. The data entry tables for these pages are shown in Figure 4. In the first table the curator enters the strain information (Figure 4A) and in the second table the curator is directed to upload a file containing gene information for genes contained in the strain (Figure 4B). The application accepts a semicolon-separated plain text file, containing values for the nine gene attributes defined in the Gene class, for batch insertion of gene information into the database. The software then stores the data in the database allowing for subsequent retrievals and updates to be performed.

#### Data modification

**Figure 5:**
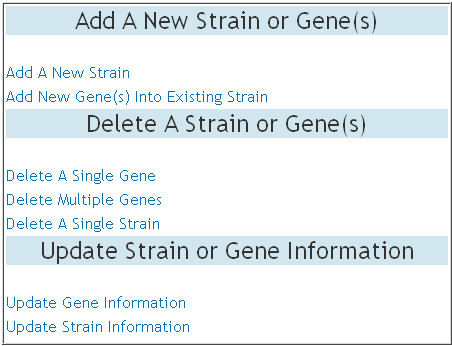
**Data Modification**. The table displayed on the web page for modifying gene and strain data. As shown in the table, modifications that can be made by the curator to gene and strain data include adding, deleting and updating gene or strain information.

**Figure 6:**
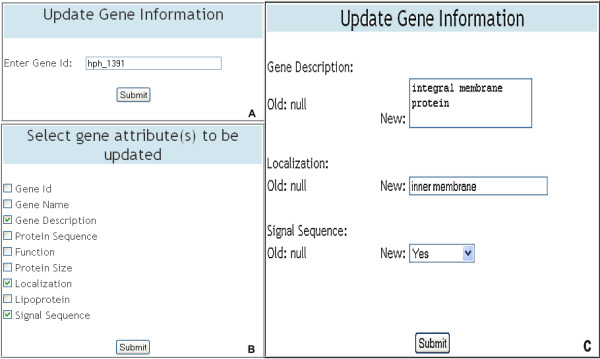
**Updating gene information**. The data entry tables displayed on the web pages for updating gene information. In the top left table (A) the gene Id of the gene whose information needs modification is entered and submitted. The table on the bottom left (B) allows the curator to select gene attributes that need to be modified. Subsequently, as shown in table (C), for each attribute selected, the gene information currently stored in the database is displayed on the left side of the table as "Old" information and on the right side of the table changes to the gene information may be entered under "New".

The DraGnET application provides web pages for assigned curators to modify genome data as well as administrator/curator user information. As shown in Figure 5, modifications that can be made to gene and strain data include adding, deleting and updating gene or strain information. The addition and deletion of single or multiple genes to strain(s) already stored in the database follows the same procedure as the addition of a strain and its associated gene set. To delete a single strain the user selects the strain to be deleted and once submitted, the strain information and all of the genes not associated with any other strain are deleted. An important part of data management is the ability to update or modify the information stored in the database, as is the case for draft genome sequences as progress is made toward gap closure and genome completion. To update gene information, the curator enters the gene Id of the gene to be updated (Figure 6A). Subsequently, the gene attributes that need to be modified are selected (Figure 6B). Once the selections are submitted the gene information currently stored in the database is displayed as "old" information and the "new" information may be entered (Figure 6C). A similar procedure is provided for updating strain information.

#### Formatting BLAST database files

BLAST functionalities for sequence similarity searches and cross strain comparisons are provided through DraGnET web-interfaces. To use these functionalities, BLAST database files for each strain stored in the database must be created through command line arguments. The command used to format BLAST database files is the similar for each strain stored in the database, having to change only the FASTA file used for BLAST database file generation. Details of this process are included in the DraGnET setup package. Once the BLAST databases are created, all BLAST functionalities offered with DraGnET are available for use.

### Data Exploration

The following functionalities are implemented through the web interface and are available for all users.

#### Quick and Advanced Search

**Figure 7:**
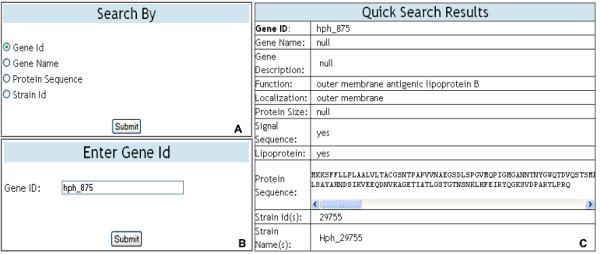
**Quick Search**. The data entry tables and results table displayed on the web pages for "Quick Search". In the top left table (A) the user selects a gene or strain search attribute. In the bottom left table (B) the user enters information for the chosen search criteria. Subsequently, the results table (C) displays information for the chosen gene or strain.

**Figure 8:**
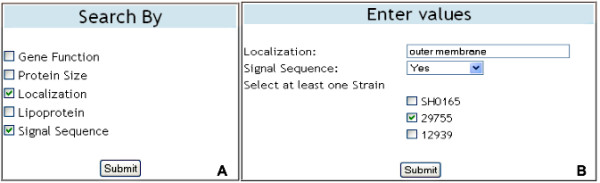
**Advanced Search**. The data entry tables displayed on the web pages for "Advanced Search". Using the table on the left (A) users may customize their search for gene information by selecting single or multiple search attributes. The table on the right (B) allows users to enter and select values for the chosen search criteria. Subsequently, a text file containing search results is available for download.

The "Quick Search" option provides users with four different search options for retrieving gene and strain information stored in the database. Searches can be performed by selecting and entering a gene Id, gene name, protein sequence, or strain Id (Figure 7A-B). When a search is performed using a gene Id, gene name or protein sequence the results are displayed in a table containing information for the chosen gene, including the strain that contains the gene (Figure 7C). Searches based upon a strain Id provide the user with strain information as well as the option to download a text file containing gene information for all of the genes contained in the chosen strain. The "Advanced Search" option allows users to search for gene information using more stringent parameters. Users can specify single or multiple gene attributes to use in the advanced search (Figure 8A). Once the attribute(s) are chosen, the user enters search criteria for each attribute chosen and selects the strain(s) they want to search (Figure 8B). If more than one strain is chosen, then the program searches for genes having the same gene identifier and chosen attributes in common with the set of strains. Search results are written to a text file that can be opened for immediate viewing or saved for future inspection.

#### BLAST Search

**Figure 9:**
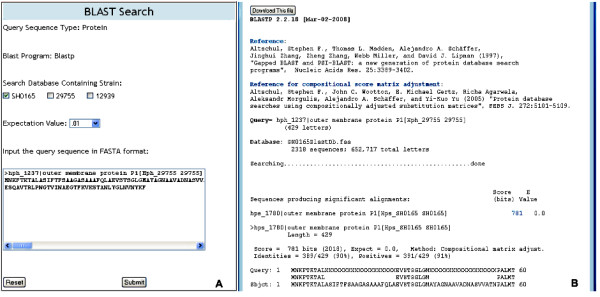
**BLAST Search**. The data entry table and results displayed on the web pages for "BLAST Search". As shown in table (A), users have the option to select a single or multiple search database(s). In this example we have three BLAST databases available for searching that represent strains SH0165, 29755 and 12939. To refine their search, users have the option to change the E-value with the default being .01. A text box is provided for users to enter a FASTA formatted protein query sequence. The results (B) are displayed to the user and are available for download.

"BLAST Search" provides users with an interface for using the protein BLAST (blastp) program for comparing protein sequences against protein sequence BLAST databases. Each strain stored in the database is used to format BLAST protein databases during the initial setup of a DraGnET project. Subsequently, the strains appear on the BLAST Search page listed under "Search Databases Containing Strains" where users have the option to select single or multiple strain databases to search against (Figure 9A). All parameters are set to NCBI defined default values; however users have the option to refine their search by changing the expectation value (E-value). Users can then input their FASTA formatted query sequence by pasting it into the query box. The output generated will include the input query sequence, the user chosen BLAST database(s) and a list of alignments between the input query sequence and the database hits. The output file is available for immediate viewing and downloading (Figure 9B).

#### Batch BLAST Search

**Figure 10:**
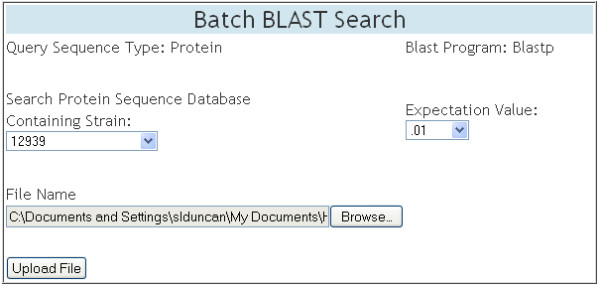
**Batch BLAST Search**. The data entry table displayed on the web page for "Batch BLAST Search". Users select a single search database from a drop down menu. In this example, the BLAST database representing strain 29755 was chosen. To refine their search, users have the option to change the E-value with the default being .01. Users then upload a text file containing FASTA formatted protein sequences which will be used as the set of query sequences. The results format is the same as "BLAST Search".

"Batch BLAST Search" is an extension of "BLAST Search" that allows text files containing multiple FASTA formatted protein sequences (including entire strains) to be uploaded for comparison against a single strain BLAST database (Figure 10). Similar to "BLAST Search", the results are written to a text file available for viewing and downloading.

#### Batch BLAST Dissimilarity Search

"Batch BLAST Dissimilarity Search" takes as input the results of "Batch BLAST Search" and extracts the gene identifiers associated with protein sequences that produced a "no hits found" result. The resulting set of genes identifiers represents genes that have no protein sequence homology to any sequences found in the selected search database. Results are written to a text file.

#### Generate FASTA Files

The "Generate FASTA Files" option automatically generates FASTA files for each strain stored in the database. When users click on the "Generate FASTA Files" button a set of files in FASTA format, one for each strain, will be available to download. Subsequently, the files can be used with other publicly available comparative analysis software tools or they can be saved as text files for use with "Batch BLAST Search".

### Case study: *Haemophilus parasuis* genome data

To demonstrate the functionalities of DraGnET we used the web application to store genomic data from three strains of *Haemophilus parasuis*, two draft genomes (strains 29755 and 12939) and a complete reference genome (strain SH0165) [46], and to perform preliminary cross strain comparisons to identify protein products common to each strain. *H. parasuis *is a bacterial pathogen that causes severe respiratory disease in swine and vaccines effective against multiple isolates are lacking [47]. Since outer membrane proteins, including lipoproteins, that are shared among the *H*. *parasuis *strains represent potential broadly protective antigens, identifying common genes is a first step toward vaccine development. Draft genome sequence data for strains 29755 and 12939 were generated using the Illumina/Solexa Genome Analyzer II platform (G. Phillips, D. Dyer, and K. Register, unpublished data). The genomes were assembled using SH0165 as a reference genome using NextGene software (State College, PA). Annotation was performed through the Institute for Genome Sciences (IGS) Annotation Engine offered by the University of Maryland, School of Medicine.

Initially, annotated genome sequence data representing the three strains were formatted for use with DraGnET by conversion to semicolon-separated files. Subsequently, information for each strain was entered and the corresponding file was uploaded and populated in the database using the application's web interface (Figure 4). Once in the database, the annotated data was available for searching and modifying. As shown in Figure 7, "Quick Search" was used to search for information related to the gene identifier "hph_875"; which returned a table with the annotated gene information. Data modification is an important functionality provided through DraGnET, especially in the case of draft genome data. To demonstrate this capability, gene information related to gene identifier "hph_1391" was selected for updating. As shown in Figure 6, the following gene attributes were selected and modified: gene description, localization, and signal sequence. Once submitted, all modifications to the data were confirmed using "Quick Search". DraGnET provides additional functionalities for preliminary analysis of draft and complete genome data. To identify protein products common to all three of the *H. parasuis *strains, "Advanced Search" and BLAST functionalities provided through the DraGnET interface were used to perform preliminary cross strain comparisons. This demonstrates the DraGnET application is ideally suited for smaller companies or academic labs that are just beginning to use next-generation sequencing for vaccine development.

## Discussion

While data from genome sequencing projects typically become publicly available through sequence repositories, the rate at which large-scale sequences information is being generated and subsequent analysis will, in many cases, delay public availability of the data. In addition, sequencing projects where proprietary data are generated are limited as to how the information can be managed and analyzed until it is ready for public reposition. This limitation emphasizes the need for software applications that provide researchers with in-house data management and analysis capabilities. While some of the features of DraGnET are provided with other applications, our software provides a user friendly in-house web application that enables researchers to manage their own unpublished or proprietary annotated draft genome data at the initial stage of development without having prior knowledge of query languages necessary for data storage, retrieval and update.

Additional features of the application include BLAST capabilities and the automatic generation of FASTA files from protein sequence data stored in the database. A web interface is provided for use of stand-alone BLAST alleviating the requirement to perform searches through command line and allowing users to search against a single strain or multiple strains as well as perform cross strain comparisons once the BLAST database files are created. DraGnET was designed to store and compare different strains from the same species; however the web interface design is generic enough to accommodate multiple organisms and their related strains. Additionally, the DraGnET software can be further developed to customize the program for specific needs.

As demonstrated in the case study, DraGnET provides researchers with an application that can be used as a first step toward data curation and analysis. Subsequently, after the data are made publicly available, more comprehensive analysis may be performed, for example by any of the aforementioned analysis software. Alternatively, the sequence data can continue to be analyzed using in-house programs, including annotation and BLAST comparisons [37,48].

Currently gene attributes selected for storage and use with DraGnET are fixed. Further development of DraGnET will include the storage of more comprehensive annotation data as well as more advanced functionalities for comparative analysis.

DraGnET currently contains draft and complete genome data from three strains of *H. parasuis *made available for collaborative research efforts. Readers are encouraged to visit the DraGnET website located at http://www.dragnet.cvm.iastate.edu and examine the functionalities of the software.

## Conclusions

New genome sequencing methods now allow multiple draft genomes to be generated, assembled, and annotated at an unprecedented rate at modest expense. Following sequencing, assembly and annotation, there is an immediate need for the data to be organized, stored, curated and formatted for comparative analysis. The DraGnET software is an ideal in-house tool that allows i.) storage and integration of annotated data generated from different annotation platforms in a database, ii.) retrieval of gene and strain information based upon basic or advanced search parameters, iii.) management of gene and strain information, iv.) generation of FASTA formatted files for all strains stored in the database, v.) sequence similarity searches using BLAST, vi.) Batch BLAST searches for cross strain comparisons and vii.) retrieval of strain specific genes based upon Batch BLAST results. The application allows for the setup of individual projects used on local machines or may be deployed through Internet (or Intranet) access for use by other researchers across different locations. To demonstrate this, we setup a DraGnET project, deployed it for Internet access, and identified potential vaccine targets in multiple strains of *H. parasuis *using preliminary cross strain comparisons.

## Availability and Requirements

• Project name: Draft Genome Evaluation Tool (DraGnET)

• Project home page: http://www.dragnet.cvm.iastate.edu

• Operating system(s): Microsoft Windows 2003 and Windows XP

• Programming language: Java

• Other requirements: JRE 1.6.0, MySQL 5.0, MySQL 5.0GUI Tools, Apache Tomcat 6.0 and Blast 2.2.18

• License: GNU GPL

## Authors' contributions

SD and RS planned wrote and tested the software. SD and GP wrote and revised the manuscript. LM assisted with the initial software design and with manuscript revisions. All authors have read and approved the manuscript.

## Supplementary Material

Additional file 1 **Source code for the DraGnET software**. This folder contains the source code for the DraGnET software. Click here for file

Additional file 2 **MySQL Database Schema**. This file contains a diagram of the MySQL database tables that are automatically created when setting up a DraGnET project. Click here for file
