# Supplementary figures and images for: DraGnET: Software for storing, managing and analyzing annotated draft genome sequence data
Source: BMC Bioinformatics. 2010 Feb 22;11:100. doi: 10.1186/1471-2105-11-100 (PMC3098051; doi:10.1186/1471-2105-11-100)

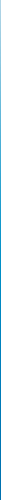

Supplement: Additional file 1 — Source code for the DraGnET software. This folder contains the source code for the DraGnET software. [file 1471-2105-11-100-S1.ZIP › DraGnETSourceCode/images/background.gif]

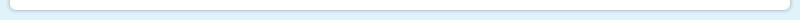

Supplement: Additional file 1 — Source code for the DraGnET software. This folder contains the source code for the DraGnET software. [file 1471-2105-11-100-S1.ZIP › DraGnETSourceCode/images/bottom.jpg]

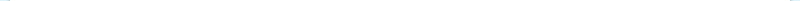

Supplement: Additional file 1 — Source code for the DraGnET software. This folder contains the source code for the DraGnET software. [file 1471-2105-11-100-S1.ZIP › DraGnETSourceCode/images/content.jpg]

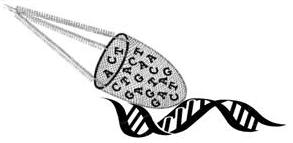

Supplement: Additional file 1 — Source code for the DraGnET software. This folder contains the source code for the DraGnET software. [file 1471-2105-11-100-S1.ZIP › DraGnETSourceCode/images/dragDNA2cpy.JPG]

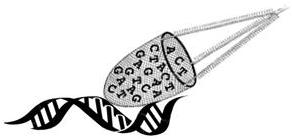

Supplement: Additional file 1 — Source code for the DraGnET software. This folder contains the source code for the DraGnET software. [file 1471-2105-11-100-S1.ZIP › DraGnETSourceCode/images/dragDNA2Rev.JPG]

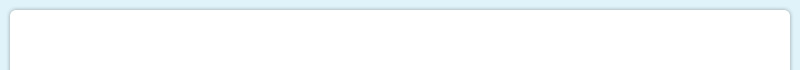

Supplement: Additional file 1 — Source code for the DraGnET software. This folder contains the source code for the DraGnET software. [file 1471-2105-11-100-S1.ZIP › DraGnETSourceCode/images/header.jpg]

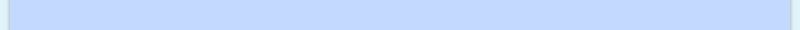

Supplement: Additional file 1 — Source code for the DraGnET software. This folder contains the source code for the DraGnET software. [file 1471-2105-11-100-S1.ZIP › DraGnETSourceCode/images/menu.jpg]

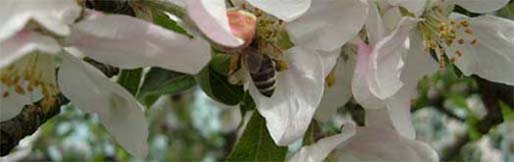

Supplement: Additional file 1 — Source code for the DraGnET software. This folder contains the source code for the DraGnET software. [file 1471-2105-11-100-S1.ZIP › DraGnETSourceCode/images/p.jpg]

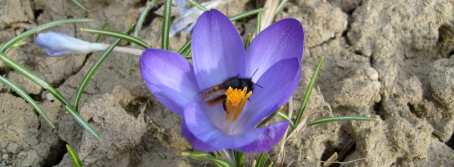

Supplement: Additional file 1 — Source code for the DraGnET software. This folder contains the source code for the DraGnET software. [file 1471-2105-11-100-S1.ZIP › DraGnETSourceCode/images/pic.jpg]

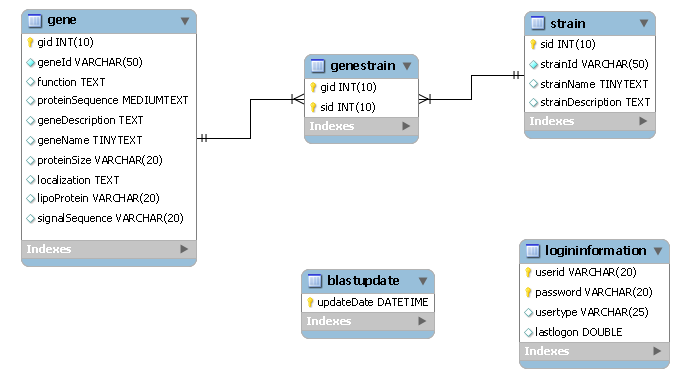

Supplement: Additional file 2 — MySQL Database Schema. This file contains a diagram of the MySQL database tables that are automatically created when setting up a DraGnET project. [file 1471-2105-11-100-S2.DOCX]
